# A meta‐analysis of perfusion parameters affecting weight gain in ex vivo perfusion

**DOI:** 10.1111/aor.14841

**Published:** 2024-08-19

**Authors:** Riley Marlar, Fuad Abbas, Rommy Obeid, Sean Frisbie, Adam Ghazoul, Ava Rezaee, Jack Sims, Antonio Rampazzo, Bahar Bassiri Gharb

**Affiliations:** ^1^ Department of Plastic Surgery Cleveland Clinic Cleveland Ohio USA

**Keywords:** ex vivo, ex vivo machine perfusion, ex vivo organ perfusion, ex‐situ perfusion, ex‐vivo perfusion, machine perfusion, perfusion, preservation, weight gain

## Abstract

**Background:**

Ex vivo machine perfusion (EVMP) has been established to extend viability of donor organs. However, EVMP protocols are inconsistent. We hypothesize that there is a significant relationship between specific parameters during EVMP and perfusion outcomes.

**Methods:**

A meta‐analysis of literature was conducted in accordance with the Preferred Reporting Items for Systematic Review and Meta‐Analysis (PRISMA) Statement. The search encompassed articles published before July 25, 2023. PubMed, Embase, and CENTRAL databases were screened using search terms “ex‐vivo,” “ex‐situ,” “machine,” and “perfusion.” Weight gain, an indicator of organ viability, was chosen to compare outcomes. Extracted variables included perfused organ, warm and cold ischemia time before perfusion, perfusion duration, perfusate flow, pressure, temperature, perfusate composition (presence of cellular or acellular oxygen carrier, colloids, and other supplements) and percent weight change. Data were analyzed using SPSS statistical software.

**Results:**

Overall, 44 articles were included. Red blood cell‐based perfusates resulted in significantly lower weight gain compared to acellular perfusates without oxygen carriers (11.3% vs. 27.0%, *p* < 0.001). Hemoglobin‐based oxygen carriers resulted in significantly lower weight gain compared to acellular perfusates (16.5% vs. 27%, *p* = 0.006). Normothermic perfusion led to the least weight gain (14.6%), significantly different from hypothermic (24.3%) and subnormothermic (25.0%) conditions (*p* < 0.001), with no significant difference between hypothermic and subnormothermic groups (24.3% vs. 25.0%, *p* = 0.952). There was a positive correlation between flow rate and weight gain (*ß* = 13.1, *R* = 0.390, *p* < 0.001).

**Conclusions:**

Oxygen carriers, low flow rates, and normothermic perfusate temperature appear to improve outcomes in EVMP. These findings offer opportunities for improving organ transplantation outcomes.

## Graphical abstract

Oxygen carriers, lower flow rates, and normothermic perfusate temperature appear to improve outcomes in ex vivo machine perfusion.

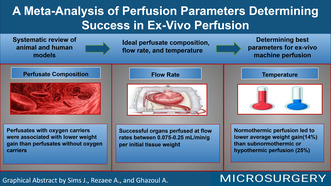

## 1 INTRODUCTION

Organ transplantation is one of the greatest accomplishments of modern medicine, offering solutions for patients with end‐stage organ failure. However, the success of transplantation depends on the quality and viability of donor organs. Ischemia–reperfusion injury (IRI) during organ procurement and storage has historically limited the preservation times of these organs, often leading to suboptimal outcomes in transplantation.^1^ The conventional method of static cold storage (SCS) has been the standard for decades but has limitations, especially in the context of metabolically active organs.^2^ In the last decade, Ex vivo machine perfusion (EVMP) has emerged as a solution to extend and improve the viability of donor organs. Recent advancements in EVMP have shown potential in addressing the shortcomings of traditional cold storage. The need for improved organ preservation techniques to reduce IRI and enhance transplant outcomes has motivated extensive research in this field.^3^

Despite the promises of ex vivo machine perfusion (EVMP), a significant challenge exists. The protocols employed in EVMP vary widely across different studies, leading to inconsistent results. Lack of consensus on critical factors including oxygen carriers, colloid choice and concentration, temperature, hematocrit, and flow rate/pressure is evident in various studies.^4^, ^5^, ^6^, ^7^, ^8^ Understanding the intricate relationship between specific perfusion parameters and perfusion outcomes is paramount to harness the full potential of EVMP. To objectively evaluate such a heterogeneous body of literature, it is essential to choose an outcome measure that is reliable, translatable to clinical outcomes, and commonly reported in machine perfusion studies. Based on our previous research^9^ and corroborating studies by other authors,^10^, ^11^, ^12^ weight gain was identified as an early clinical indicator of organ viability directly correlating with histologic changes within the tissue.^9^, ^10^, ^11^, ^12^

We hypothesized that there is a significant relationship between the variation in specific perfusion parameters during EVMP and the outcomes of organ perfusion, specifically with regard to weight gain. Determining the relative importance of these parameters can allow optimization of EVMP protocols.

## 2 MATERIALS AND METHODS

### 2.1 Study design

A meta‐analysis of literature was conducted in accordance with the Preferred Reporting Items for Systematic Review and Meta‐Analysis (PRISMA) Statement.

The search encompassed all articles published before July 25, 2023. PubMed, Embase, and CENTRAL were screened using search terms “ex‐vivo,” “ex‐situ,” “machine,” and “perfusion.” All references mentioned in the identified original articles were reviewed to identify additional non‐indexed literature. A study was deemed eligible for inclusion if it specified perfusion duration and perfusate composition, and if it evaluated the change in organ weight after ex vivo machine perfusion. Weight gain, as an early clinical indicator of organ viability, directly correlating with histologic changes, was chosen to facilitate comparison between organs.^9^, ^10^, ^11^, ^12^ Exclusion criteria included systematic reviews, meta‐analyses, conference abstracts, studies without weight data, and studies in which experimental or pharmacologic intervention could affect the success of the perfusion. Articles were excluded if they reported weight gain data in a format that prevented the calculation of percentage weight gain, such as using wet/dry ratio or providing grams gained without specifying a starting or final weight.

Articles were uploaded to Covidence for title and abstract screening and full text review to determine eligibility. Eligible studies underwent data extraction. Extracted variables included species, organ, warm and cold ischemia time before initiation of perfusion, duration of perfusion, perfusate flow, pressure, temperature, composition of perfusate (presence of oxygen carrier, colloids, other supplements), hematocrit, and weight change.

Perfusate temperature was classified as hypothermic (0–12°C), subnormothermic (13–34°C), or normothermic (35–38°C).^13^ Perfusates were classified by their oxygen carrier, such as red blood cell (RBC‐based), hemoglobin‐based oxygen carrier (HBOC), or acellular without oxygen carrier (AWOC). HBOC‐201 (Hemopure®, HbO2 Therapeutics LLC, Souderton PA) was the only formulation of HBOC present in studies that met inclusion criteria.^5^, ^9^, ^14^, ^15^, ^16^ Hematocrit analysis was conducted for experiments utilizing red blood cell‐based perfusates, to establish a correlation with weight gain. When red blood cells were mixed with solutions, such as Steen solution or saline, the resulting diluted hematocrit was calculated. Flow rates were frequently reported in mL/min. We converted this measurement to mL/min/g of starting tissue weight. This was done to facilitate comparison between organs from different species, therefore different sizes. Cold ischemia time was recorded as the length of time the organ was preserved on ice between procurement and ex vivo machine perfusion. Warm ischemia time was recorded as the time the organ was kept at room temperature between procurement and ex vivo machine perfusion. The source of supplemental albumin was recorded for each study. Colloid concentrations were recorded for each experimental group.

Bias evaluation was performed by three reviewers who independently assessed the experimental methods and study design. If there was disagreement, the decision was deferred to the senior author for determination of eligibility.

### 2.2 Data analysis

Variables are reported as mean and standard deviation (mean ± SD). For continuous data, Student's *t*‐tests or analysis of variance (ANOVA) with or without Welch modification, followed by Tukey post‐hoc pairwise comparison, were performed as appropriate. In correlation analysis, Pearson's correlation coefficient was computed, and the results were reported as the beta coefficient (*ß*), correlation coefficient (*R*), and *p*‐value (*p*). Data analysis was conducted using IBM SPSS Statistics version 26.0.0.0. *p* values <0.05 were considered statistically significant.

## 3 RESULTS

### 3.1 Study selection

**FIGURE 1:**
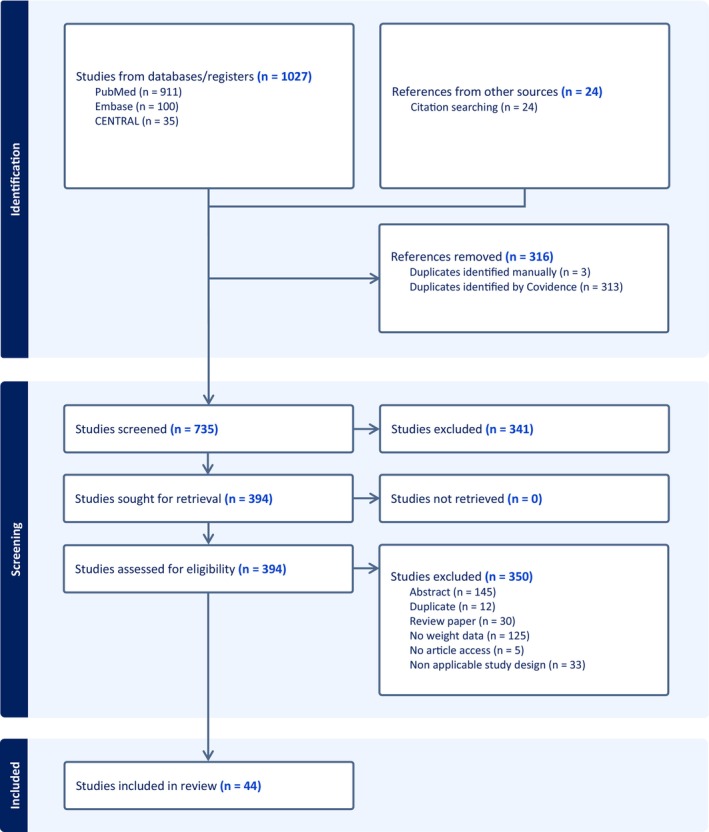
PRISMA study flow diagram. [Color figure can be viewed at wileyonlinelibrary.com]

A total of 1027 studies were retrieved. Forty‐four studies satisfied the inclusion and exclusion criteria and were included.^2^, ^4^, ^6^, ^7^, ^8^, ^9^, ^10^, ^14^, ^15^, ^17^, ^18^, ^19^, ^20^, ^21^, ^22^, ^23^, ^24^, ^25^, ^26^, ^27^, ^28^, ^29^, ^30^, ^31^, ^32^, ^33^, ^34^, ^35^, ^36^, ^37^, ^38^, ^39^, ^40^, ^41^, ^42^, ^43^, ^44^, ^45^, ^46^, ^47^, ^48^, ^49^, ^50^, ^51^ The PRISMA flow diagram is shown in Figure 1.

### 3.2 Characteristics of included studies

A total of 44 studies were included in this systematic review. These studies consisted of 100 experimental groups. Experimental groups within each study were analyzed independently. Experimental groups were weighted by sample size. Sample size was defined as the number of individual organs within each experimental group. A summary of the characteristics of the included studies and basic experimental parameters is presented in Table 1.

**TABLE 1:** Summary of outcomes included in analysis.

	Organ	Pressure	Flow	Duration	Hematocrit	Albumin	Weight change	*p*‐value	Citation
	*n*	mm Hg	mL/min/g	h	%	g/L	%		
Oxygen carrier									
RBC	208	55.1	0.41	22.1	19.0	46.9	11.3 ± 13.9	Reference	[4, 5, 6, 10, 15, 17, 20, 21, 22, 24, 34, 35, 36, 38, 39, 41, 43, 45, 50, 52]
HBOC	36	53.5	0.12	13.2	13.3^a^	45.20	16.5 ± 6.5	0.299	[5, 9, 14, 15, 16]
AWOC	312	37.1	0.71	11.3	N/A	57.5	27.0 ± 23.0	<0.001	[2, 7, 8, 14, 18, 19, 21, 23, 27, 28, 29, 30, 31, 32, 35, 36, 37, 40, 43, 44, 46, 47, 48, 49, 51]
Albumin source									
BSA	122	59.9	0.35	5.60	20.3	56.0	17.7 ± 21.5	Reference	[2, 8, 14, 17, 27, 29, 43, 44]
HSA	123	41.8	0.69	35.4	18.6	57.0	14.4 ± 17.6	0.091	[5, 6, 7, 10, 20, 21, 24, 28, 37, 41, 48]
Temperature									
Hypothermic	196	28.6	0.48	13.4	22.0	30.10	24.3 ± 20.9	<0.001	[7, 17, 18, 19, 23, 27, 28, 30, 31, 32, 35, 36, 37, 40, 46, 47, 51]
Subnormothermic	131	50.3	0.90	10.1	18.8	72.18	25.0 ± 21.0	<0.001	[2, 8, 14, 15, 17, 29, 30, 38, 39, 44, 48, 49, 50]
Normothermic	229	55.1	0.47	20.3	18.9	49.49	14.6 ± 18.3	Reference	[4, 5, 6, 9, 10, 16, 17, 20, 21, 22, 24, 25, 34, 35, 36, 41, 43, 45, 52]

*Note*: All results reported as averages. hypothermic ≤12°C; subnormothermic 13–34°C; normothermic ≥35°C.

Abbreviations: AWOC, acellular without oxygen carrier; BSA, bovine serum albumin; HBOC, hemoglobin‐based oxygen carrier; HSA, human serum albumin; *n*, number of organs; N/A, not applicable; RBC, red blood cell.

^a^ Expected hematocrit calculated from hemoglobin concentration.

### 3.3 Species and organs

There were five different species represented in these experiments: pig (*n* = 299), rat (*n* = 107), dog (*n* = 66), human (*n* = 55), and rabbit (*n* = 29). Six different organs were studied: vascularized composite allografts (*n* = 162), kidney (*n* = 142), heart (*n* = 113), liver (*n* = 71), lung (*n* = 53), and pancreas (*n* = 15).

### 3.4 Weight gain by organ

Kidneys experienced greater of weight gain than other organs (37.7% ± 25.1%, *p* < 0.001). Lungs (18.5% ± 11.2%), hearts (15.6% ± 12.4%), pancreas (15.0% ± 0%), livers (14.6% ± 21.2%), and vascularized composite allografts (12.6% ± 14.8%) had similar weight gain (*p* ≥ 0.325).

### 3.5 Perfusate temperature

The least amount of weight gain was observed in the normothermic group (14.6% ± 18.3%) compared to the subnormothermic (25.0% ± 21.2%, *p* < 0.001) and hypothermic (24.3% ± 20.9%, *p* < 0.001) groups. Weight gain in subnormothermic and hypothermic groups was not significantly different (*p* = 0.958).

### 3.6 Perfusate composition

Perfusion pressure was the same in RBC‐based perfusates and HBOC perfusates (55 ± 29 mm Hg vs. 54 ± 27 mm Hg, *p* = 0.944). AWOC perfusates flowed at lower perfusion pressures than perfusates with oxygen carriers (37 ± 24 mm Hg, *p* < 0.002). Weight gain was lower in RBC‐based perfusates compared to AWOC perfusates (11.3% ± 13.9% vs. 27.0% ± 23.0%, *p* < 0.001). HBOC perfusates also resulted in lower weight gain compared to AWOC perfusates (16.5% ± 6.4% vs. 27% ± 23.0%, *p* = 0.006). There was no difference in weight gain between HBOC perfusates and RBC‐based perfusates (16.5% ± 6.4% vs. 11.3% ± 13.9%, *p* = 0.299).

### 3.7 Hematocrit

**FIGURE 2:**
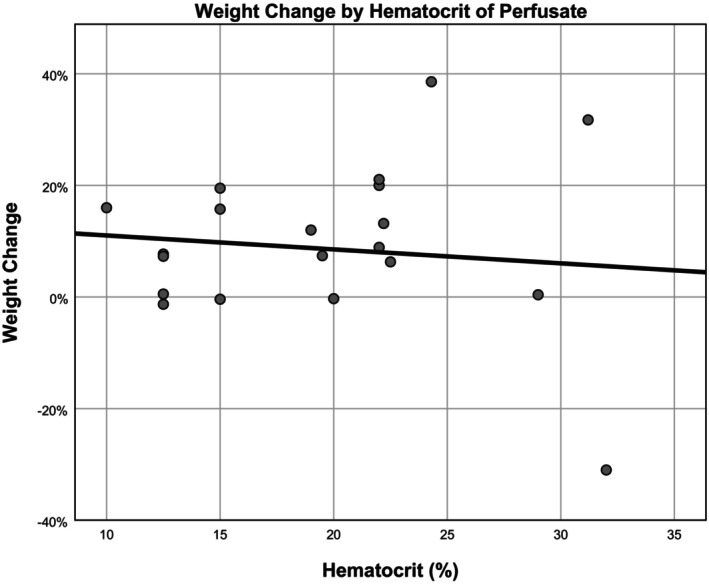
Organ weight change by hematocrit of perfusate. This figure is a scatter plot illustrating the relationship between organ weight change and the hematocrit level of the perfusate.

The average perfusate hematocrit in RBC‐based perfusates was 19% ± 6.3% (range 10–32%). There was no correlation between weight gain and hematocrit (*ß* = −0.296, *R* = 0.122, *p* = 0.177) (Figure 2).

### 3.8 Flow rate and pressure during perfusion

**FIGURE 3:**
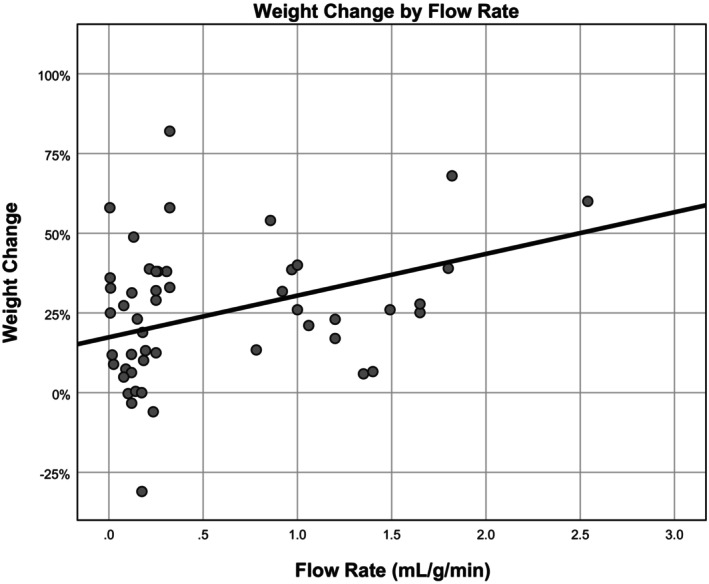
Organ weight change by flow rate of perfusate. This figure is a scatter plot showing the relationship between organ weight change and the flow rate of the perfusate.

The average flow rates were as follows: lungs (1.49 ± 0 mL/g/min), kidneys (0.92 ± 0.84 mL/g/min), pancreas (0.78 ± 0 mL/g/min), livers (0.72 ± 0.44 mL/g/min), hearts (0.50 ± 0.48 mL/min/g), and vascularized composite allografts (0.09 ± 0.06 mL/min/g). Flow rate was highest for AWOC perfusates (0.71 ± 0.72 mL/min/g, *p* = 0.013). Flow rate was not different between RBC‐based perfusates (0.41 ± 0.46 mL/min/g) and HBOC perfusates (0.12 ± 0.04 mL/min/g, *p* = 0.367). Flow rate was highest for subnormothermic perfusions (0.9 ± 0.9 mL/min/g, *p* < 0.001). Flow rate was not different between normothermic (0.47 ± 0.47/ mL/min/g) and hypothermic perfusions (0.48 ± 0.48 mL/min/g, *p* = 0.923). There was a positive correlation between flow rate and weight gain (*ß* = 13.1, *R* = 0.390, *p* < 0.001) (Figure 3). There was no correlation between perfusate pressure and weight gain (*ß* = 0.019, *R* = 0.028, *p* = 0.545), and no correlation between flow rate and pressure (*ß* = 4.086, *R* = 0.113, *p* = 0.062).

### 3.9 Cold and warm ischemia times

**FIGURE 4:**
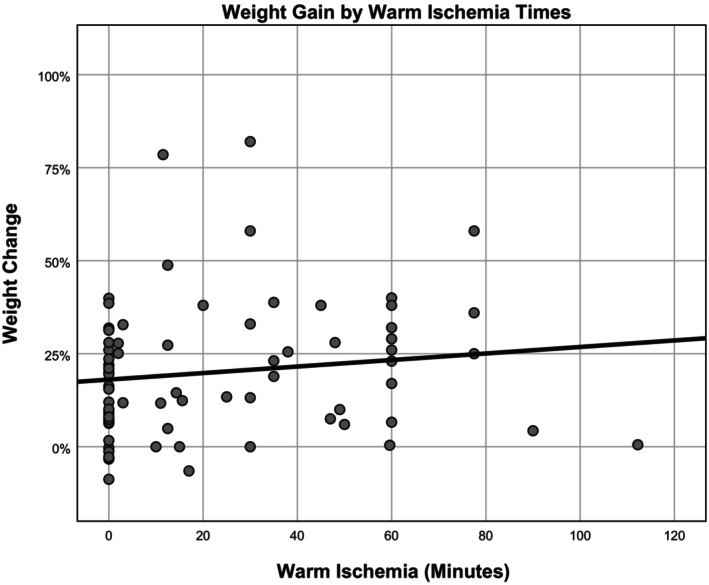
Organ weight change by warm ischemia time. This figure is a scatter plot depicting the relationship between organ weight change and warm ischemia time.

Weight gain was not correlated with cold ischemia time (81 ± 161 min, range 0 to 1080, *p* = 0.656). Warm ischemia time (21 ± 25 min, range 0–112) was positively correlated with weight gain (*ß* = 0.088, *R* = 0.127, *p* = 0.009) (Figure 4).

### 3.10 Duration of perfusion

**FIGURE 5:**
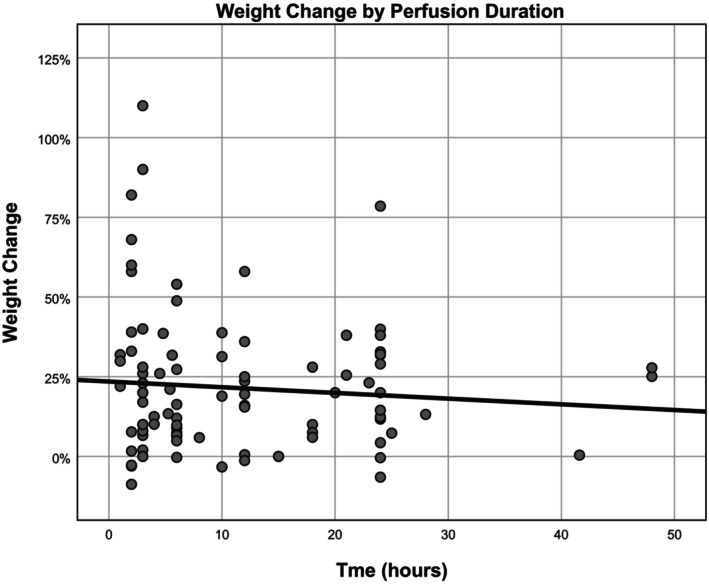
Organ weight change by duration of perfusion. This figure is a scatter plot that presents the relationship between organ weight change and the duration of perfusion.

The average duration of the perfusion was 15.5 ± 26.9 h (range 1–168). Despite the large range, 94% of experiments were 24 h or less. There was a negative correlation of weight gain and duration of perfusion (*ß* = −0.233. *R* = 0.303, *p* < 0.001) (Figure 5).

### 3.11 Colloids

Weight gain was not significantly different in experiments with albumin as primary colloid (15.8% ± 17.2%) as compared to hydroxyethyl starch (HES) (16.5% ± 15.3%, *p* = 1.0). Albumin and HES had less weight gain than mannitol (32.8% ± 17.4%, *p* < 0.001). Albumin, HES, and mannitol lead to less weight gain than experiments with no colloids (46.5% ± 26.4%, *p* < 0.015). The majority (*n* = 145, 65%) of experiments were supplemented with additional albumin to increase the oncotic pressure of the perfusate. Supplemental albumin was either human serum albumin or bovine serum albumin. There was no significant difference in organ weight gain between perfusates supplemented with bovine serum albumin (17.7% ± 21.5%) or human serum albumin (14.4% ± 17.6%, *p* = 0.091). There was no correlation between albumin concentration and weight gain (*ß* = 0.043, *R* = 0.073, *p* = 0.225). There was no correlation between HES concentration and weight gain (*ß* = 0.269, *R* = 0.189, *p* = 0.079).

## 4 DISCUSSION

Organ preservation through machine perfusion stands as a critical domain in modern medicine, offering promising solutions for enhancing organ viability and reducing ischemia–reperfusion injury (IRI) during transplantation. In this meta‐analysis, we reviewed the diverse perfusion parameters that could affect the success of ex vivo perfusion.

Success of ex vivo perfusion can be measured by different parameters, depending on the organ being perfused. Lungs are evaluated by perfusate gases and biochemical properties, gross anatomy, ventilation, and oxygenation capacity.^53^ In contrast, livers are assessed by bile production, perfusate acid/base homeostasis, pressure/flow parameters, and homogeneous perfusion with “soft” parenchymal consistency.^54^ In order to facilitate comparison across multiple studies, each with their own diverse markers of viability, we chose weight gain as a non‐invasive, objective measure across all organs. In our previous experimental studies, one of the earliest markers of perfusion failure is weight gain, which reflects the culmination of a cascade of events, encompassing endothelial injury, heightened fluid permeability, and interstitial edema.^9^, ^10^, ^11^, ^12^ Organs are affected differently by weight gain. Hearts are commonly reported to undergo myocardial edema in ex vivo machine perfusion, although the impact is unclear, with hearts of different levels of edema performing equivalently.^55^ For example, Cobert found that weight increase of canine hearts perfused at 5°C for 10 h with glucose‐supplemented Celsior (which lacks an oncotic agent) were higher than hearts perfused with University of Wisconsin containing hydroxyethyl starch (31% increase vs. 3% weight loss, *p* < 0.005), although both groups maintained similar oxygen consumption, lactate accumulation, and regional flow distributions in the ventricles throughout the preservation period.^23^ In contrast to hearts, lungs seem to be more sensitive to weight increase. Per the Toronto ex vivo lung perfusion protocol, donor lungs that exhibit signs of pulmonary edema should undergo further testing before transplantation, as this can be an indicator of poor post‐transplant outcomes.^56^, ^57^ Kosaka et al. sustained that lung weight gain after 40 min of perfusion correlates with PaO_2_/FiO_2_, peak inspiratory pressure, shunt ratio, and transplant suitability at 2 h of perfusion, with successful lungs having a cutoff of less than 12 g of weight gain at 40 min of perfusion.^58^ In a subsequent study, the same group showed that lung weight gain for cases considered suitable for transplantation was 7% (−9% to 35%), whereas the weight gain of non‐suitable lungs was 74% (range, 26%–117%).^59^ An organ less sensitive to weight gain, the kidney, can be transplanted at higher weight gain. Wilson et al. showed that kidneys with 30% weight gain after hypothermic perfusion can be transplanted with no difference in secondary outcome measures.^60^ Although in this experiment, the edematous kidneys showed more pronounced signs of vascular disturbances with endothelial disruption as well as inflammatory infiltration. While studying normothermic preservation of porcine limbs, Meyers et al. determined weight gain correlates directly with histological evidence of muscle injury (muscle injury scores were already higher than baseline at 5% weight gain), and can facilitate assessment of limb suitability for transplantation.^9^

Conflicting reports in the literature have called into question the optimal perfusion temperature. In a direct comparison of subnormothermic perfusion and normothermic perfusion, Arni et al. concluded that subnormothermic perfusion of lungs lead to lower inflammatory markers as well as increased oxygenation of the lungs.^61^ Some countries, such as the Netherlands, have introduced hypothermic machine perfusion for all deceased donor kidneys.^62^ This meta‐analysis revealed reduction in weight gain during normothermic perfusion compared to subnormothermic and hypothermic approaches, which might be attributed to the restoration of aerobic metabolism, potentially minimizing or circumventing ischemic injury.^63^ Counterintuitively, the perfusions of longer duration tended to have lower weight gain. In this study, this could be explained by the duration of normothermic perfusions being longer than the duration of hypothermic and subnormothermic perfusions. In general, the longer duration of machine perfusion is associated with organ weight gain,^9^, ^55^, ^64^, ^65^ as despite the maximal effort to preserve physiologic conditions, the perfusion does not mimic all the complexities of body fluids and their homeostatic regulation. At some point, a combination of ischemic and metabolic injury occurs causing interstitial edema and weight gain. Successful machine perfusion can delay the organ failure and even stimulate organ conditioning and weight loss initially.^66^ Weight loss during normothermic perfusions despite longer duration would support that these perfusion conditions were better than the hypothermic and subnormothermic perfusions that exhibited greater weight gain despite shorter duration. Current research does not suggest weight loss observed in ex vivo machine perfusion causes a decrease in the function of the organ.^24^, ^52^

Literature has suggested that HBOC is capable of rapidly scavenging NO, which results in vasoconstriction and hypertension.^67^, ^68^, ^69^ Interestingly, this meta‐analysis did not identify a significant difference between HBOC‐based perfusates and RBC‐based perfusates with regard to weight gain, perfusate pressure, and, indirectly, vascular resistance. This is supported by the results of Figueroa et al. who compared HBOC and RBC‐based perfusates in porcine limbs, and determined that both perfusate types had similar outcomes in a 24 h perfusion.^15^ This meta‐analysis found that both HBOC‐based perfusates and RBC‐based perfusates lead to less weight gain than acellular solutions without oxygen carriers (AWOC) perfusates such as Steen, University of Wisconsin, and Krebs–Henseleit solutions, emphasizing the impact of oxygen carriers in ex vivo perfusion. An unsurprising result, considering that tissue hypoxia activates gene transcription factors like nuclear factor kappa B that produce proinflammatory cytokines contributing to edema.^70^

The hematocrit levels in ex vivo perfusates are frequently debated, with some studies proposing an optimal level of 20%.^71^ And other studies, like Steen, determined that 15% hematocrit is optimal.^72^ In Steen's study, the authors rationalized that a higher hematocrit would require more perfusion pressure to maintain adequate flow. In contrast, our review consisted of several studies that maintained a low weight with a higher hematocrit.^24^, ^41^ In our analysis, all experiments with RBC‐based perfusates were within 10–32% hematocrit, and we determined no effect of hematocrit on weight gain. These results imply that factors other than hematocrit may exert a more pronounced influence on the weight gain observed in ex vivo perfusion.

We confirmed a relationship between flow rate and weight gain. All organs that had less than 5% of weight gain were perfused at a flow rate between 0.075 mL/min/g and 0.25 mL/min/g flow rate per initial organ weight.^14^, ^17^, ^23^, ^24^, ^32^ These findings echo associations reported by other researchers, like Kosaka, who identified a correlation between lung weight gain and flow rate.^58^ In our study, flow rates exceeding the threshold of 0.25 mL/min/g appear to be correlated with weight gain, possibly due to factors such as shear stress on red blood cells, endothelial cells, and mechanical hemolysis.^16^, ^73^, ^74^ Evaluation of tissue ischemia during ex vivo perfusion will need to incorporate outcomes of organs after transplantation. Studies aiming to identify the minimal perfusion rate necessary to prevent tissue ischemia have been conducted, such as Quader's experiment in 2020.^74^ In Quader's study, they were not able to prevent tissue ischemia in a rat heart perfused at 0.3 mL/g/min; however, they attributed this to an insufficient amount of oxygen carrying molecules within their perfusate. The results of our study support this result, as perfusates with oxygen carriers experienced less weight gain than the solutions without oxygen carriers. Kidneys suffered greater weight gain than other organs in ex vivo perfusion. However, 132 of 142 total kidneys were perfused with acellular perfusates, which might have affected this significantly increased weight gain. Further analysis should incorporate each organ's unique energy requirements, as this can tailor recommendations for each organ.

We did not find a correlation between pressure and flow rate, a surprising result considering the logical relationship between these two variables, as well as the many protocols that choose to maintain a certain pressure by adjusting flow rates.^9^ We hypothesize that this is because pressure measurements are influenced by the perfusion machine circuitry. In a 2014 study of the accuracy of invasive arterial pressure monitoring,^75^ they concluded that using a 20 gauge versus 18 gauge catheter for invasive blood pressure monitoring leads to a significant difference in pressure readings due to damping of the arterial pressure signal between the vessel and the pressure transducer. This effect is further enhanced with longer tubing and larger diameter catheters.^75^ Furthermore, many perfusion experiments monitor pressure with T fittings and other connectors between the pressure transducer and the artery.^8^, ^50^ Because of these factors, the pressure reading is likely skewed and cannot be accepted as an accurate measurement of actual intra‐arterial pressure. Researchers must keep in mind these inconsistencies when interpreting data from other studies and applying concepts to their own experiments.

To decrease long waitlist times for organ recipients, some authors are interested in exploring the possibility of using organs with extended warm ischemia times. In an experiment by Charles in 2017,^76^ they successfully transplanted a porcine lung after 2 h of warm ischemia time. Our analysis of perfusion experiments has shown a correlation with warm ischemia times and increased weight during perfusion. Although, two studies with human limbs had less than 5% weight gain as well as an ischemia time of 90 and 112 min.^4^, ^7^ It is clear that warm ischemia is not beneficial to the success of the perfusion, but studies highlighted above suggest that some perfusion protocols can have a rehabilitative effect on the organs, and allow them to tolerate increased warm ischemia times.

Another consideration in the context of human perfusion studies is that organs declined for transplant, and consequently available for perfusion studies, may be more edematous at the onset. We agree that in certain clinical scenarios limited weight gain might not correlate with organ function. Ours^41^ and others experience^10^, ^24^ show that edematous organs could undergo either weight loss or weight gain during ex vivo perfusion. In the former scenario, the organ is reconditioned during perfusion and the high albumin perfusate extracts the edema from the interstitium, usually with improved organ function. In the second case, the organ has been damaged beyond repair and would gain weight despite its edematous state, with increased resistance and loss of function.

Eshmuminov et al.^24^ perfused edematous human livers that were denied for transplant for 7 days. The human livers were severely injured and heterogeneous with respect to cold and warm ischemia times, underlying liver diseases, donor age, duration of intensive care unit treatment, among other parameters. During machine perfusion livers exhibited signs of viability such as consuming oxygen, producing bile, and clearing lactic acid, while losing 31% of their weight. Okamoto et al.^10^ found that the donor lungs with a lung weight >800 g may have extra water due to the sequelae of donor management, adrenergic storm, and volume overload. This extra water was considered a possible target of fluid removal in ex vivo lung perfusion via its osmolality‐gradient mechanism. The average lung weight change in these suitable cases (donor lung weight: 800–1280 g) was 20 g. In contrast, donor lungs with lung weight ≥1280 g had significantly lower recovery rate with higher lung weight change during perfusion (+151 g). They hypothesized that the intrapulmonary fluid contents in this group was beyond the capacity of fluid removal in ex vivo perfusion or because of injured vasculature of perfused lungs.

Cold ischemia time did not correlate with weight gain. Eshmuminov et al. perfused human livers with up to 597 min of cold ischemia time with weight loss during the experiment.^24^ However, no heart, lung, or kidney perfusions that exhibited less than 5% weight gain in our study reported cold ischemia times. It is difficult to determine limits of cold ischemia time through this study, but the lack of correlation with cold ischemia times and weight gain, as well as the example highlighted above, suggest that some amount of cold ischemia is permissible.

Bovine Serum Albumin (BSA) is often favored in scientific investigations because of lower cost and greater availability compared to Human Serum Albumin (HSA). Although these molecules are generally similar, there are subtle differences between the two. For example, HSA is more thermally stable and more hydrophobic than BSA.^77^ Some studies suggest that the origin of albumin can affect outcomes in EVMP.^78^ This meta‐analysis indicated comparable weight gain in experiments supplemented with HSA and BSA. These results imply that the use of BSA in future studies might serve to reduce the overall cost of perfusion procedures without compromising the outcomes. Our study found no difference between perfusion experiments supplemented with albumin or hydroxyethyl starch (HES). Other conclusions, such as the lack of correlation between weight gain and colloid concentration, and the inferiority of mannitol as a primary colloid, should be further studied. Perfusates have a diverse composition of ingredients that contribute to the total oncotic and osmotic pressure. It is difficult to attribute the effects to a single ingredient based on the concentration. This type of analysis would be better suited using an osmometer or oncometer,^79^ which can account for the interaction between multiple substrates as they contribute to the oncotic or osmotic pressure of a solution.

## 5 LIMITATIONS

The main limitation of this study lies in the inherent nature of a systematic review. Our data collection and analysis are dependent on the quality standards of perfusion protocols and reported outcomes of the studies extracted. Perfusion solutions were frequently supplemented with additives that we were not able to account for during the analysis. The intrinsic difference of each organ possibly influences experimental outcomes and weight gain. Nonetheless, a major strength of our article lies in the inclusion of a wide range of studies from multiple organs which contributes to the large sample size in our results.

## 6 CONCLUSIONS

This meta‐analysis shed light on several critical aspects influencing organ viability during ex vivo perfusion. Perfusates with oxygen carriers delivered at normothermic temperatures experienced the least amount of weight gain. Perfusates supplemented with hydroxyethyl starch, bovine serum albumin, and human serum albumin lead to comparable outcomes. Increased warm ischemia time was associated with higher weight gain, while cold ischemia time did not have a pronounced impact. Hematocrits between 12.5 and 32% did not appear to affect weight gain. Flow rates in machine perfusion with less than 5% organ weight were between 0.075 and 0.25 mL/min/g of starting tissue weight. Despite these insights, the study's limitations call for further prospective research with standardized methodologies to validate these findings and establish robust protocols for ex vivo perfusion, thereby improving transplant outcomes and expanding the horizons of biomedical research.

## AUTHOR CONTRIBUTIONS

Concept/design: RM, FA, BBG; Data analysis/interpretation: RM, FA, RO, BBG; Drafting article: RM; Critical revision of article: RM, AR, BBG; Approval of article: AR, BBG; Statistics: RM; Data collection: RM, FA, RO, SF, AG, AR, JS.

## FUNDING INFORMATION

No funding was received for this study.

## CONFLICT OF INTEREST STATEMENT

The authors have nothing to disclose.
